# Linking Glycation and Glycosylation With Inflammation and Mitochondrial Dysfunction in Parkinson’s Disease

**DOI:** 10.3389/fnins.2018.00381

**Published:** 2018-06-07

**Authors:** Paula A. Q. Videira, Margarida Castro-Caldas

**Affiliations:** ^1^UCIBIO, Departamento Ciências da Vida, Faculdade de Ciências e Tecnologia, Universidade NOVA de Lisboa, Caparica, Portugal; ^2^CDG & Allies – Professionals and Patient Associations International Network (CDG & Allies – PPAIN), Faculdade de Ciências e Tecnologia, Universidade NOVA de Lisboa, Caparica, Portugal; ^3^Research Institute for Medicines (iMed.ULisboa), Faculty of Pharmacy, Universidade de Lisboa, Lisbon, Portugal

**Keywords:** Parkinson’s disease, mitochondrial dysfunction, inflammation, glycation, glycosylation, aging

## Abstract

Parkinson’s disease (PD) is the second most common neurodegenerative disorder, affecting about 6.3 million people worldwide. PD is characterized by the progressive degeneration of dopaminergic neurons in the *Substantia nigra pars compacta*, resulting into severe motor symptoms. The cellular mechanisms underlying dopaminergic cell death in PD are still not fully understood, but mitochondrial dysfunction, oxidative stress and inflammation are strongly implicated in the pathogenesis of both familial and sporadic PD cases. Aberrant post-translational modifications, namely glycation and glycosylation, together with age-dependent insufficient endogenous scavengers and quality control systems, lead to cellular overload of dysfunctional proteins. Such injuries accumulate with time and may lead to mitochondrial dysfunction and exacerbated inflammatory responses, culminating in neuronal cell death. Here, we will discuss how PD-linked protein mutations, aging, impaired quality control mechanisms and sugar metabolism lead to up-regulated abnormal post-translational modifications in proteins. Abnormal glycation and glycosylation seem to be more common than previously thought in PD and may underlie mitochondria-induced oxidative stress and inflammation in a feed-forward mechanism. Moreover, the stress-induced post-translational modifications that directly affect parkin and/or its substrates, deeply impairing its ability to regulate mitochondrial dynamics or to suppress inflammation will also be discussed. Together, these represent still unexplored deleterious mechanisms implicated in neurodegeneration in PD, which may be used for a more in-depth knowledge of the pathogenic mechanisms, or as biomarkers of the disease.

## Parkinson’s Disease

Parkinson’s disease (PD) is the second most common age-related neurodegenerative disease, clinically characterized by typical motor symptoms such as resting tremor, rigidity, bradykinesia, gait, and balance dysfunction that result in near total immobility and strongly impair patients’ quality of life (Chaudhuri et al., 2006; Thomas and Beal, 2007; Jankovic, 2008). Currently, it is well accepted that several non-motor symptoms are also a key component of PD. These symptoms include hyposmia, constipation, hallucinations, depression, anxiety, sleep dysfunction, apathy, and dementia, and some of them may even arise in the pre-motor phase of the disease (Zis et al., 2015; Poewe et al., 2017).

Aging is the main risk factor for developing PD, a disease that affects about 1% of the population above 60 years (Tysnes and Storstein, 2017). There is yet no objective test or biomarker for PD, so the ultimate diagnosis of PD is *post-mortem*. The main neuropathological hallmarks are the progressive degeneration of pigmented dopaminergic neurons within the *Substantia nigra pars compacta* (SNpc) resulting into dramatic reduction of striatal dopamine, and α-synuclein-containing inclusions, called Lewy bodies, in the surviving neurons (Dauer and Przedborski, 2003; Tysnes and Storstein, 2017).

Epidemiological studies revealed that approximately 90% of PD cases have a sporadic origin, which may be caused by unknown environmental factors together with genetic susceptibility. The remaining 10% of PD cases represent familial forms of the disease (Thomas and Beal, 2007). Out of the six gene mutations responsible for monogenic PD, two are accountable for autosomal dominant (AD) PD forms (*SNCA* and *LRRK2*) and the remaining four for autosomal recessive (AR) PD (*PARK2*, *PINK1*, *DJ-1*, and *ATP13A2*) (Klein and Westenberger, 2012). The main mutations involved in familial forms of PD are summarized in **Table 1**.

**Table 1:** Genes and loci associated with autosomal recessive (AR) or autosomal dominant (AD) Parkinsonism.

PARK locus	Gene	Full gene name	Inheritance	Disease onset
*PARK1/4*	*SNCA*	Alpha-synuclein	AD	Early-onset parkinsonism with common dementia
*PARK2*	*PARKIN*	Parkin	AR	Early-onset parkinsonism, slowly progressive
*PARK3*	Unknown	Unknown	AD	Late onset parkinsonism
*PARK5*	*UCHL1*	Ubiquitin C-terminal hydrolase	AD	Early- and late-onset parkinsonism
*PARK6*	*PINK1*	PTEN induced putative kinase 1	AR	Early-onset, slowly progressive parkinsonism
*PARK7*	*DJ-1*	DJ-1 (parkinsonism associated deglycase)	AR	Early-onset parkinsonism
*PARK8*	*LRRK2*	Leucine rich repeat kinase 2	AD	Late-onset parkinsonism
*PARK9*	*ATP13A2*	ATPase 13A2	AR	Early-onset parkinsonism with Kufor-Rakeb syndrome
*PARK10*	Unknown	Unknown	Unclear	Late-onset parkinsonism
*PARK11*	*GIGYF2*	GRB10 interacting GYF protein 2	AD	Late-onset parkinsonism
*PARK12*	Unknown	Unknown	X-linked inheritance	Late-onset parkinsonism
*PARK13*	*HTRA2*	HtrA serine peptidase 2	AD	Unclear
*PARK14*	*PLA2G6*	Phospholipase A2 group VI	AR	Early-onset dystonia parkinsonism
*PARK15*	*FBX07*	F-box protein 7	AR	Early-onset parkinsonism with pyramidal syndrome
*PARK16*	Unknown	Unknown	Unclear	Late-onset parkinsonism
*PARK17*	*VPS35*	VPS35, retromer complex component	AD	Late-onset parkinsonism
*PARK18*	*EIF4G1*	Eukaryotic translation initiation factor 4 gamma 1	AD	Late-onset parkinsonism
*PARK19*	*DNAJC6*	DNAJ heat shock protein family (Hsp40) member C6	AR	Early-onset parkinsonism
*PARK20*	*SYNJ1*	Synaptojanin 1	AR	Early-onset parkinsonism
*PARK21*	*TMEM230*	Transmembrane protein 230	AD	Unclear
*PARK22*	*CHCHD2*	Coiled-coil-helix-coiled-coil-helix domain containing 2	AD	Unclear
*Gaucher’s locus*	*GBA*	Glucocerebrosidase	AD	Late-onset parkinsonism

### Evidence for Mitochondrial Dysfunction in Parkinson’s Disease

Mammalian mitochondria contain 2 to 10 molecules of mtDNA, a double-stranded circular genome of about 16.6 kb that encodes 22 transfer RNAs (tRNA), 2 ribosomal RNAs (rRNA), and 13 polypeptides (Schapira, 1994). However, it still depends on nuclear enzymes to replicate and translate its 13 polypeptides encoding genes, all of which generate a small proportion of subunits of the respiratory chain complexes. For example, complex I is composed of about 40 protein subunits but only seven of those are encoded by mtDNA (Schapira, 1994). The remaining subunits of this multimeric complex are nuclear encoded and must be imported to mitochondria, and properly assembled in the inner mitochondrial membrane. The mitochondrial respiratory chain constitutes the site of oxidative phosphorylation responsible for NADH and FADH2 oxidation, concomitantly with the translocation of protons from the matrix to the intermembrane space, establishing an electrochemical gradient commonly known as mitochondrial membrane potential (ΔΨm). The electrochemical gradient of protons drives the action of ATP synthase, reducing molecular oxygen and synthesizing ATP. This step is fundamental in aerobic metabolism, and constitutes the main provider of ATP at the final stage of cellular respiration (Schapira, 1994). During oxidative phosphorylation electrons may leak from the electron transport chain and may react with oxygen generating reactive oxygen species (ROS), which under normal circumstances are removed by antioxidant agents in the mitochondria (Keane et al., 2011). The absence of a protective envelope favors mtDNA damage by ROS due to the proximity to the main source of production of these reactive molecules. Additionally, mtDNA is highly vulnerable to damages due to inefficient DNA repair mechanisms and to an absence of a protective histone coating (Schapira et al., 1990; Schapira, 1994). This vulnerability increases with aging when the endogenous antioxidant defense mechanisms tend to be down-regulated. Such oxidative injury to mitochondria and other cellular structures in the brain accumulates with time leading to several deleterious effects associated with age-related neurodegenerative disorders like PD (Perry et al., 2009; Schapira, 2012). This highly reactive, reduced species of oxygen are responsible for the oxidative damage of lipids, proteins and nucleic acids including the mitochondrial components themselves predisposing to apoptotic cell death.

Mitochondria are highly dynamic organelles, that continually fuse and divide, and the balance between fusion and fission determines the size, shape, number and function of these organelles (Bose and Beal, 2016). Damaged and/or obsolete mitochondria are selectively removed by a quality control mechanism known as mitochondrial autophagy or mitophagy. Coordination between clearance of damaged mitochondria and mitochondrial biogenesis is essential in the maintenance of a healthy mitochondrial pool and cellular homeostasis. Thus, mitophagy is essential in mitochondrial turnover regulation, by adjusting the amount of organelles accordingly to metabolic requirements of cells. It is generally accepted that mitochondrial biogenesis depends upon fusion events, while fission is assumed to isolate damaged organelles that can then be targeted for degradation.

Importantly, the fine-tuned regulation of mitophagy seems to be impaired in PD, which is not an odd observation, since this mechanism is regulated by parkin and by the serine/threonine protein phosphatase and tensin homolog (PTEN)-induced putative kinase 1 (PINK1), and mutations on these proteins are known to be related with rare familial forms of PD.

### Parkin, PINK1, and α-Synuclein in Parkinson’s Disease

Half of familial PD cases are associated with a wide variety of loss-of-function mutations in *PARK2* gene, encoding for parkin, a constituent of a E3 ubiquitin ligase complex. These mutations also account for about 20% of the cases with early onset, indicating loss-of-function pathogenic mechanisms in PD (Thomas and Beal, 2007; Martin et al., 2011; Klein and Westenberger, 2012). Parkin is an interesting protein involved in the modulation of different aspects of mitochondrial turnover. In fact, parkin, together with PINK1, regulates mitophagy and maintains mitochondrial homeostasis (Martin et al., 2011; Song et al., 2013). Importantly, parkin also regulates mitochondrial biogenesis by modulating the levels of parkin interacting substrate (PARIS), a transcriptional repressor of PGC-1α (Shin et al., 2011). peroxisome proliferator-activated receptor gamma coactivator-1α (PGC-1α) is an important co-activator of mitochondrial transcription factors and thus a master regulator of mitochondria biogenesis (Shin et al., 2011). Accordingly, deletion of PGC-1α gene renders dopaminergic neurons more vulnerable, whereas its over-expression is neuroprotective in experimental models of PD (Zheng et al., 2010; Jiang et al., 2016). Mutations in *PINK1* gene also cause autosomal recessive PD with early onset, being the first gene identified that suggested that impaired mitochondrial function was involved in PD pathogenesis (Silvestri et al., 2005). The majority of the mutations identified in *PINK1* are non-sense or missense mutations that affect the serine/threonine kinase domain, suggesting that loss of kinase function may be an important part of PINK1-induced pathogenesis in PD (Klein and Westenberger, 2012; Song et al., 2013). Parkin and/or PINK1 loss of function may lead to the accumulation of injured mitochondria, which will contribute to increased production of oxidative stress that may possibly underlie PD pathogenesis.

On the other hand, only a small number of mutations have been reported for *SNCA* gene, including missense mutations (e.g., A53T, A30P, A53E, and E46K mutations), duplications and triplications, all of them implicated in familial PD (Nuytemans et al., 2010). The *SNCA* gene encodes the α-synuclein protein, whose normal function of is still not fully understood, although evidence indicates that it is a pre-synaptic protein involved in neurotransmitter release (Burre et al., 2010; Nemani et al., 2010). However, over-expression of this protein and its gain-of-function pathological mutations promote the formation of oligomeric species and fibrils that are considered the main toxic species triggering deleterious mechanisms in PD (Conway et al., 2000; Outeiro et al., 2009). In fact, aggregated α-synuclein is the primary fibrillary component of Lewy bodies (Lee and Trojanowski, 2006) and oxidative and nitrosative stresses promote its aggregation, which in turn can damage mitochondria, contributing to further oxidative stress and neuron degeneration.

### Oxidative Stress in Parkinson’s Disease

The etiology of PD still remains unknown, although several mechanisms leading to the neurodegenerative process, associated with dopaminergic neuron loss have been proposed. These include mitochondrial complex I dysfunction, impairment of ATP production, oxidative stress, neuroinflammation, endoplasmic reticulum (ER) stress and aberrant proteolytic degradation. Among these, mitochondrial dysfunction seems to play a key role in PD, since it may lead to over-production of ROS, inflammatory responses, and activation of cell death pathways (Mizuno et al., 1989; Przedborski et al., 2004).

Indeed, mitochondrial impairment caused by mutations in genes linked to familial PD, together with data from human *post-mortem* tissue indicate that impaired mitochondrial function, increased oxidative stress and deficient anti-oxidant capacity are common pathological mechanisms implicated in the etiology of both familial and sporadic PD cases (Dauer and Przedborski, 2003; Perry et al., 2009). Accordingly, *post-mortem* observations of PD patients’ brains revealed a decreased activity of mitochondrial complex I in the SNpc (Mizuno et al., 1989; Schapira et al., 1990; Moore et al., 2005). Moreover, additional studies also revealed mitochondrial complex I deficits in platelets and skeletal muscle of PD patients (Yoshino et al., 1992; Dexter and Jenner, 2013). Mitochondrial complex I inhibitors, such as 1-methyl-4-phenyl-1,2,3,6-tetrahydropyridine (MPTP), and its toxic metabolite (1-methyl-4-phenylpyridinium, MPP^+^) induce a cascade of events, leading to neuropathological features of the disease in humans and animals (Chun et al., 2001; Nicotra and Parvez, 2002; Dauer and Przedborski, 2003; Castro-Caldas et al., 2012), further reinforcing the involvement of mitochondrial dysfunction in PD pathogenesis.

Studies using PD brain mitochondria indicate that oxidation of complex I catalytic subunits is the mechanism responsible for its reduced function and disassembly (Keeney et al., 2006). Interestingly, oxidative and nitrosative stress can also inactivate parkin by inducing post-translational protein modifications and misfolding with the subsequent accumulation of PARIS, and may also accelerate α-synuclein aggregation, which together may account for mitochondrial dysfunction (West and Maidment, 2004; Dawson and Dawson, 2010; Chakraborty et al., 2017). Additionally, several evidences support the idea that ATP depletion and ROS overproduction occurs soon after injection of the mitochondrial complex I inhibitor MPTP in mice (Dauer and Przedborski, 2003; Castro-Caldas et al., 2012; Moreira et al., 2017). Although complex I inhibition elicited by MPP^+^ reduces ATP production and increases ROS production within dopaminergic neurons, mounting evidence indicates that rather than killing the cells, these changes are triggering the activation of cell death molecular pathways, which underlie the demise of damaged neurons (Cleeter et al., 1992; Przedborski et al., 2004). These damages are thought to be irreversible and can induce neurodegeneration of the nigrostriatal dopaminergic system. In parallel, it has also been described an impaired antioxidant ability of SN neurons in PD in part due to lower levels of reduced glutathione (Dexter and Jenner, 2013).

### Protein Quality Control Mechanisms in Parkinson’s Disease

Protein quality control mechanisms is the way by which cell monitors proteins to ensure that they are appropriately folded, and prevents overload of aberrant proteins, and formation of toxic aggregates, allowing cells to cope with environmental stresses. The ubiquitin-proteasome system (UPS) is the major non-lysosomal protein degradation pathway within cells (Ciechanover and Kwon, 2017). The UPS is critical for degradation of misfolded intracellular proteins, through the regulation of protein turnover and cellular response to stress (Ciechanover and Kwon, 2017). Impairment of UPS has already been reported in patients with familial PD, namely with mutations in genes linked to quality control mechanisms, such as parkin and ubiquitin C-terminal hydrolase-L1 (Kitada et al., 1998; Leroy et al., 1998; Dawson and Dawson, 2003; Martin et al., 2011). Sporadic PD patients also display impaired proteasomal activity in the SNpc (McNaught and Jenner, 2001). Impairment of UPS in PD triggers a cycle of cell-damaging events including accumulation of misfolded proteins, aggregation of α-synuclein and mitochondrial dysfunction.

Another important quality control pathway is autophagy. In light of results published in the literature, there is still controversy about the role of autophagy in PD. On one hand, it has been shown that inhibition of the autophagic flux by MPTP underlies the effect of this toxin on neuronal survival (Liu et al., 2013). Indeed, an efficient activation of autophagy/mitophagy as a response to mitochondrial damage or neuronal injury has a clear pro-survival role, and this process has been reported as being deregulated in some PD models (Zhu et al., 2007; Chu, 2011; Menzies et al., 2015; Rosa et al., 2017). On the other hand, the active metabolite of MPTP, MPP^+^, in culture cells may be an inducer of autophagy as a cell death pathway (Zhu et al., 2007; Wong et al., 2011). Nonetheless, fine tune regulation of autophagy seems to be impaired in PD, contributing to the accumulation of toxic species and misfolded proteins. The disproportional increase in the concentration of misfolded proteins and toxic species has critical consequences on the normal functioning of cells, in particular of organelles such as mitochondria and ER.

Although several ER quality control mechanisms exist, chronic cellular stress may disrupt the mechanisms of protein folding with concomitant increase of misfolded proteins. Accumulation of these abnormal proteins in the lumen of the ER activates the adaptive signaling pathway designated by unfolded protein response (UPR). This mechanism is controlled by the ER resident chaperone glucose-regulated protein-78 (GRP78) that regulates the activity of 3 important sensors of stress: protein kinase RNA-like ER kinase (PERK), inositol requiring enzyme-1 (IRE1), and activating transcription factor-6 (ATF6) (Mercado et al., 2013; Tsujii et al., 2015). UPR works primarily to restore homeostasis and lessen ER stress by arresting protein translation, increasing folding ability, enlargement of ER membrane and initiation of ER-associated degradation (ERAD) pathway (Mercado et al., 2013; Tsujii et al., 2015). Interestingly, ER stress has been gaining increasing importance in PD research (Mercado et al., 2013; Tsujii et al., 2015). In fact, it was demonstrated activation of the PERK pathway in SN of *post-mortem* PD brains (Hoozemans et al., 2007). Accordingly, a downstream partner of the PERK pathway, the C/EBP-homologous protein (CHOP), is activated upon 6-hydroxydopamine (6-OHDA) administration (Holtz and O’Malley, 2003), while ablation of CHOP is protective against 6-OHDA toxicity (Silva et al., 2005). Besides PERK, the IRE1 and ATF6 branches of the UPR have also been implicated in experimental models of PD (Mercado et al., 2013). Accordingly, *in vivo* and *in vitro* studies demonstrated that toxins that induce PD also induce ER stress, and *vice versa*. Interestingly, recently it was shown that stereotaxic injection at the level of SN of the potent ER stressor tunicamycin, an inhibitor of the first steps of the *N*-glycosylation pathway, can be used as a stress rodent model for PD (Cóppola-Segovia et al., 2017). These data collectively suggest that in PD the UPR is up-regulated, and this may account for the accumulation of misfolded proteins and aggregation of α-synuclein and subsequently to neuronal loss, possibly linking aberrant glycosylation with neuropathological events.

### Immune System and Parkinson’s Disease

Besides mitochondrial impairment and oxidative stress, activation of glial cells and neuroinflammation also play an important role in the progression of the neurodegenerative process in PD (Hirsch et al., 2003; Hald and Lotharius, 2005). Glia play a key role in brain homeostasis, continuously monitoring the neuronal microenvironment, contributing to the endogenous antioxidant defense mechanisms, as well as in supplying trophic factors to neurons (McGeer and McGeer, 2008).

Microglia are brain-resident myeloid cell population, normally present in the central nervous system (CNS) in a surveillant non-polarized state but rapidly become activated in the presence of neuronal injuries (Hald and Lotharius, 2005; McGeer and McGeer, 2008). Activation of microglia generally comprises morphological alterations, expression of cell surface markers release of pro-inflammatory cytokines, up-regulation of pro-inflammatory enzymes, particularly cyclooxygenase-2 (COX-2) and inducible nitric oxide synthase (iNOS), leading to the synthesis of prostaglandins and nitric oxide (NO), and phagocytosis of degenerating neurons and debris (Vijitruth et al., 2006; Pattarini et al., 2007). Thus, once activated by injured neurons microglia can itself become a donor source of toxic factors causing injuries to nearby neurons, in a process designated by neuroinflammation. These neuronal injuries, in turn, will induce additional microglial activation and may be an important process that drives and exacerbates the progressive neurodegeneration after the initial stimulus.

Hence, chronic inflammation seems to play a substantial role in the neurodegenerative pathway underlying PD as well as in different PD models (Hebert et al., 2003; Hirsch et al., 2003; Vijitruth et al., 2006; Pattarini et al., 2007). Microglia are thought to present a cell protective anti-inflammatory phenotype in early stages of the disease, however, due to the chronic nature of PD they tend to switch to an activated pro-inflammatory subtype at latter stages of disease progression (Tang and Le, 2016). In these circumstances where the inflammatory process is sustained and when the levels of inflammatory challenge exceed a threshold, the death of neurons and the neuroinflammation potentiate each other in a vicious manner (Gao et al., 2011; Hirsch et al., 2013).

The involvement of the immune system in the pathogenesis of PD, has been reinforced by observation of reactive microglia, as well as pro-inflammatory soluble mediators and enzymes in PD patients’ SNpc (Mogi et al., 1994; Mosley et al., 2012; Kannarkat et al., 2013). We and others have also showed that a sustained inflammatory reaction by activated microglia is also recapitulated in genetic and toxin-based experimental models of the disease (Hebert et al., 2003; Vijitruth et al., 2006; Pattarini et al., 2007; Rosa et al., 2018). Accordingly, increased expression of anti-inflammatory mediators, including interleukin 1 beta (IL-1β) and COX-2, and decreased expression of inflammation resolution proteins such as annexin-A1, were found in MPTP treated mice brain (Hebert et al., 2003; Vijitruth et al., 2006; Pattarini et al., 2007; Rosa et al., 2018). The beneficial effects of non-steroid anti-inflammatory medication, such as ibuprofen and aspirin, in PD further reinforce the contribution of neuroinflammation in dopaminergic neuron cell death and disease progression (Wang Y.D. et al., 2016; Tentillier et al., 2016; Naeem et al., 2017).

However, controversy still exists regarding the onset of neuroinflammation in PD. On one hand, some authors suggest that glial cells are effectively involved in the early stages of PD and their activation status is maintained throughout the course of disease progression (Halliday and Stevens, 2011). On the other hand, others claim that it is unlikely that glia activation initiates neuron loss in PD, but quite possibly exacerbates and sustains the neurodegenerative process. In turn, animal models indicate microglia activation in early stages, while specific their specific inhibition led to a significant protection against MPTP toxicity (Wu et al., 2002; Halliday and Stevens, 2011). Nonetheless, even if these are early events in the degenerative process, a sustained glial activation occurs in parallel with dopaminergic cell loss and is still evident in long-term treatment with MPTP and in the SNpc of human PD (Hebert et al., 2003; Hirsch et al., 2003; Vijitruth et al., 2006; Pattarini et al., 2007).

Interestingly, recent studies have shown that dopaminergic neurons display major histocompatibility complex (MHC) class I molecules and these are involved in presentation of intracellular digested proteins on the neuronal membrane that are recognized by CD8^+^ cytotoxic T lymphocytes (Cebrián et al., 2014). Remarkably, MHC class I molecules expressed at dopaminergic neurons surface are up-regulated in presence of activated microglia or other pro-inflammatory markers, such as interferon-gamma (IFN-γ) related to unusually high levels of oxidative stress (Cebrián et al., 2014). Up-regulation of MHC class I may initiate T cell mediated responses leading to neurotoxicity. Recently, it was suggested that parkin and PINK1 could mediate mitochondrial antigen presentation, repressing this pathway in dysfunctional organelles (Matheoud et al., 2016). In PD decreased parkin activity leads to increased accumulation of dysfunctional mitochondria, as well as increased antigen presentation at the surface of these organelles, triggering the recruitment and activation of microglia and peripheral infiltrating inflammatory cells. Mitochondria antigen presentation pathway dependent on PINK1 and parkin, provides an elegant way to connect mitochondrial dynamics and immunologic pathways in PD.

On the other hand, MHC II molecules at the surface of microglia and T lymphocytes were found to be deeply implicated in the inflammatory process in PD. Accordingly, abundant MHC class II positive microglia and CD4^+^, CD8^+^ T cells were found in the SN of PD patients, and microglia activity was confirmed to be related to the degeneration severity as well as PD progression (Cebrián et al., 2014; Martin et al., 2016). The contribution of immune mechanisms in PD is also corroborated by the observation that ablation of MHC class II renders mice more resistant to MPTP toxicity (Martin et al., 2016).

The infiltration of T cells and direct access to dopaminergic neurons is facilitated by a lymphatic system in the CNS that may be responsible for the transport of periphery immune cells into the brain (Louveau et al., 2015). Additionally, blood brain barrier (BBB) dysfunctions, and increased permeability have already been shown in PD patients, allowing circulating inflammatory T cells to access the brain (Kortekaas et al., 2005; Mosley et al., 2012; Sweeney et al., 2018). Concomitantly with the neurodegenerative process, reactive microglia secrete pro-inflammatory mediators, such as tumor necrosis factor alpha (TNF-α), IL-1β, and IL-6, or present antigens to CD4^+^ T cells by the MHC-II pathway (More et al., 2013; Cebrián et al., 2014). Interestingly, abnormal populations of T cells were not only found in CNS but also in peripheral blood from PD patients (Chen et al., 2016). Together these immune reactions triggered by different cell types result in increased secretion of pro-inflammatory cytokines in cerebrospinal fluid (CSF) or blood (More et al., 2013; Santiago and Potashkin, 2014). Thus, CNS infiltration of immune cells associate the peripheral immune system with the progression of PD (Mosley et al., 2012; Kannarkat et al., 2013). Additionally, PD patients showed increased levels of oxidative stress, decreased healthy mitochondrial population and accumulation of damaged mitochondria in circulating neutrophils (Vitte et al., 2004), favoring the hypothesis of systemic mitochondrial defects in PD, and indicating a possible link between mitochondrial dysfunction and inflammation. The observation of periphery immune cells in CNS of PD patients goes beyond the local involvement of microglia and neuroinflammation, but also associates the whole immune system in the progression of this disease.

Together these observations indicate the importance of central and peripheral immunity reactions in PD, keeping the current question whether inflammation is involved in triggering the disease, or occurs instead as a consequence. Recently, autoimmune diseases or peripheral infections are considered by some authors as a risk factor for the development of PD (Witoelar et al., 2017), in a current view of the disease as being a multisystem pathology and not only confined to the CNS.

## Glycation in Parkinson’s Disease

### AGE and RAGE in Parkinson’s Disease

**FIGURE 1:**
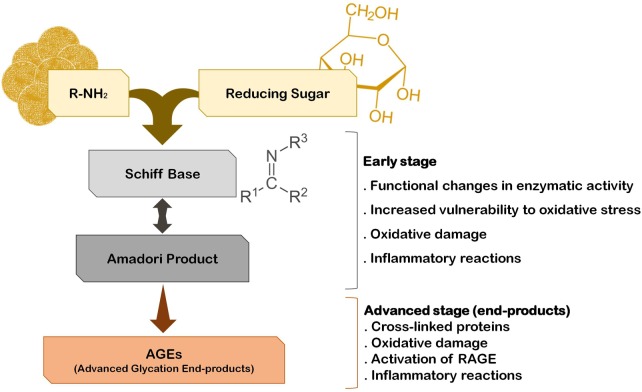
The pathway of protein glycation and AGE formation. Endogenous glycating agents include glucose, fructose, galactose, mannose, ribose, and reactive triose intermediates of energy metabolism, glyoxal and MGO. The formation of AGEs occurs spontaneously by non-enzymatic reactions in a process designated by Maillard reaction. The early stage of this reaction usually occurs rapidly (hours to days), while the advanced stage of AGE formation is thought to take weeks to months to occur.

The fact that the vast majority of PD cases are sporadic suggests that several environmental factors trigger the onset and progression of this disease. In fact, epidemiological studies indicate that PD incidence is related with exposure to pesticides, herbicides, and heavy metals. Interestingly, he risk for developing PD can also be associated with the quality of diet and metabolism. On one hand, a diet rich in fresh fruit, vegetables and fish is associated with low risk to develop PD (Gao et al., 2007). On the other hand, although some controversy still exists, it is thought that diabetes and altered glucose metabolism are strongly associated with PD, and major risk factors for the disease (Becker et al., 2008; Driver et al., 2008; Lu et al., 2014). A major consequence of diabetes is glucose metabolism imbalance and subsequent hyperglycemia, that lead to biochemical abnormalities that may trigger and/or aggravate PD progression (Vicente Miranda et al., 2016). In fact, it was demonstrated that patients that develop diabetes before PD onset tend to present more severe motor and non-motor symptoms and faster disease progression (Schwab, 1960; Arvanitakis et al., 2007; Cereda et al., 2012). Accordingly, abnormal glucose tolerance and hyperglycemia have been detected in the majority of PD cases (Lipman et al., 1974; Sandyk, 1993). Glucose and its by-products have the ability to react with amino groups, following the Maillard reaction ultimately giving rise to advanced glycation end-products (AGEs) that severely impact the function of target proteins (Yamagishi et al., 2015) (**Figure 1**). This reaction is designated by glycation, that is the glycosylation of proteins, lipids, and nucleic acids, that occurs spontaneously without being catalyzed by any enzyme. Due to its deleterious effects on biochemical targets, glycation is an important player in cellular aging (Ulrich and Cerami, 2001; Li et al., 2012; Salahuddin et al., 2014). Low amounts of AGEs are constitutively formed under normal conditions but their production is significantly augmented under conditions of hyperglycemia and oxidative stress. Accordingly, a glucose rich diet can increase up to 34-fold the generation of AGEs in the brain, particularly in the SNpc (Uchiki et al., 2012). In fact, it is now well accepted that the glycolytic pathway is not harmless reaction sequence, and its partial inhibition has been linked to increased life span in many animal experiments (Fontana et al., 2010). Methylglyoxal (MGO) is a by-product of glycolysis but can also be formed by the catabolism of serine and threonine or by lipid peroxidation. Interestingly, PD patients CSF seems to have a specific metabolomic signature that reflects alterations in glycation or glycosylation, oxidative stress and innate immunity and that may be explored as new early-stage disease biomarkers (Trezzi et al., 2017). Specifically, elevated fructose levels were found in the CSF of PD patients, which like other reducing sugars, can react with proteins through the Maillard reaction (glycation). Thus, fructose may also indicate an early event of pathological accumulation of AGEs in PD (Trezzi et al., 2017). However, both these monosaccharides may also act as ROS scavengers shifting oxidative phosphorylation to glycolysis, as a protective mechanism activated in early stages of the disease.

Advanced glycation end-products generation is a time-dependent irreversible process that leads to the accumulation and/or aggregation of proteins due to cross-linking between AGE-modified peptides. Interestingly, AGE-modified cell proteins and abnormal glycated mitochondrial proteins are implicated in mitochondria-induced oxidative stress and inflammation (Rosca et al., 2005; Rabbani and Thornalley, 2008), whereas oxidative stress seems to exacerbate the formation of AGEs (Baynes and Thorpe, 2000), eliciting a positive loop of oxidative damage in the brain. Together these AGE-dependent mechanisms may underlie, at least in part, the neurodegenerative process in PD (**Figure 1**). This is sustained by several studies indicating the presence of AGEs in the periphery of Lewy bodies, SNpc, cerebral cortex and amygdala of PD patients’ brains, suggesting that protein glycation is part of PD pathogenic mechanisms (Dalfó et al., 2005; Kurz et al., 2011). Importantly, it has even been suggested that AGEs might trigger Lewy body formation prior to the onset of PD, since they were found in Lewy bodies in incidental Lewy body disease patients’ brains (Münch et al., 2000; Dalfó et al., 2005). Glycation was also reported in several models of parkinsonism, namely the MPTP model of PD (Teismann et al., 2012; Viana et al., 2016; Vicente Miranda et al., 2017). Interestingly, in toxin-based animal models of PD the depletion of dopaminergic neurons in the SNpc is aggravated in animals that were concomitantly submitted to a high fat diet regimen (Bousquet et al., 2012; Ma et al., 2015), suggesting that this diet may increase the susceptibility of animal to PD-inducing drugs. Moreover, mice expressing A30P mutant α-synuclein fed with a high-fat diet showed earlier onset of the motor symptoms and α-synuclein aggregation (Rotermund et al., 2014), further corroborating the contribution of AGEs for PD development and/or progression.

**FIGURE 2:**
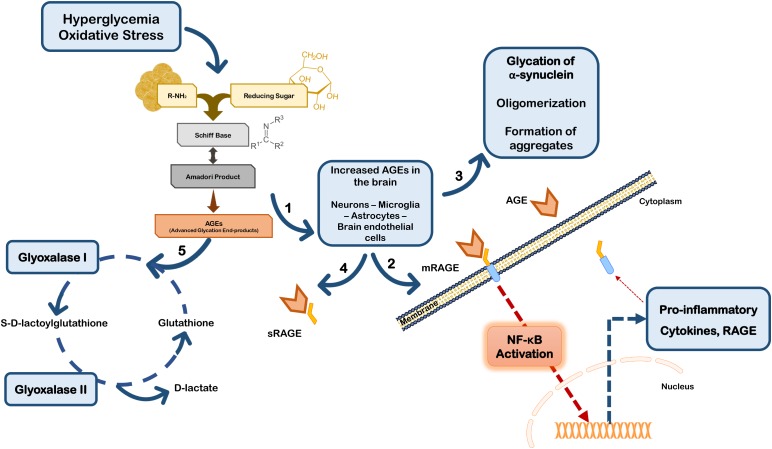
Glycation in Parkinson’s disease. Hyperglycemia and oxidative stress may up-regulate the Maillard reaction with increased formation of AGEs in brain cells (1). AGEs can either bind to membrane RAGE (mRAGE) at the cell surface of microglia and/or neurons, triggering NF-κB activation and subsequent expression of pro-inflammatory mediators and RAGE (2). Glycation can also directly affect the function of proteins, namely α-synuclein favoring its accumulation and aggregation (3). On the other hand, circulating AGEs may bind to soluble RAGE receptors (sRAGE) that do not control any intracellular pathway, acting as a neuroprotective strategy (4). Additionally, cells are endowed with efficient endogenous systems for detoxication against AGEs, namely glyoxalase-I and -II that constitute the main systems responsible for the metabolization of AGEs in a pathway dependent on glutathione (5).

Besides their direct effects on proteins, AGEs may trigger a cell response through binding to the receptor for advanced glycation end products (RAGEs). RAGE is a multi-ligand receptor of the immunoglobulin superfamily of cell surface molecules with a crucial role in the CNS in neuroinflammation, oxidative stress and neurotoxicity (Ding and Keller, 2005). In the CNS RAGE is found in neurons, microglia, astrocytes, and brain endothelial cells (Ding and Keller, 2005; Deane et al., 2012; Teismann et al., 2012; Gasparotto et al., 2017). Typically, after ligand binding RAGE induces the activation of the transcription factor nuclear factor-kappa B (NF-κB) (Bierhaus et al., 2001; Angelo et al., 2014; Tóbon-Velasco et al., 2014) (**Figure 2**). RAGE is expressed as both full-length membrane localized (mRAGE), as well as a soluble (sRAGE) isoform that lacks the transmembrane domain. Whereas mRAGE is the signaling isoform, sRAGE circulates in blood and body fluids. However, sRAGE maintains the ability to bind circulating ligands, but since it lacks the intracellular signaling domain, it does not trigger any cellular reactions (Ding and Keller, 2005; Alexiou et al., 2010; Yan et al., 2010; Kalea et al., 2011). Therefore, competing for the ligands with mRAGE acting as a decoy for ligands, sRAGE has cytoprotective effects (Yan et al., 2010; Aldini et al., 2013) (**Figure 2**). By controlling the levels of circulating AGEs, sRAGE regulates RAGE intracellular signaling, thereby avoiding over-expression of inflammatory mediators (Ding and Keller, 2005). Therefore, in PD the scenario may involve accumulation of RAGE ligands due to environmental cell conditions that favor up-regulated biosynthesis and/or impaired endogenous clearance systems.

Additionally, RAGE expression may be up-regulated by its own ligands, since the human RAGE gene has two NF-κB responsive elements on its promoter. Thus, ligand-induced RAGE activation triggers its own up-regulation and perpetuates inflammation and neurodegeneration (Ding and Keller, 2005; Tóbon-Velasco et al., 2014). In fact, RAGE activation may induce increased secretion of TNF-α which in turn, can to up-regulate cellular RAGE expression via NF-κB (Tanaka et al., 2000; Sriram et al., 2004). Interestingly, strong evidence indicates that TNF-α is involved in early and late stages in the immune pathophysiology of PD (McCoy et al., 2011), further suggesting that RAGE-inducing inflammation through NF-κB activation is implicated in PD progression. Conflicting findings on RAGE biology subsist in PD studies, however, it has been described that PD patients’ brains have increased expression of RAGE paralleled with AGEs accumulation, and that RAGE activation was linked to oxidative stress (Dalfó et al., 2005; Ding and Keller, 2005; Sathe et al., 2012). Additionally, RAGE up-regulation and activation was also demonstrated in sub-acute MPTP, rotenone and 6-OHDA mice models of PD (Teismann et al., 2012; Abdelsalam and Safar, 2015; Gasparotto et al., 2017). On the other hand, ablation of RAGE protects primary dopaminergic neurons against MPP^+^ induced toxicity (Teismann et al., 2012), and selective inhibition of RAGE prevented dopaminergic denervation and locomotory and exploratory deficits induced by 6-OHDA in rats (Gasparotto et al., 2017). Importantly, RAGE polymorphisms were associated increased susceptibility or protection against PD in Chinese Han population (Gao et al., 2014). Together these data strongly implicate RAGE expression and activation in PD, and are corroborated by the findings in several experimental models of the disease, further reinforcing the involvement of this receptor in PD pathologic mechanisms.

The intracellular signaling pathways induced upon RAGE activation depend on the ligands that bind to it and the on the cell type where it is expressed. Besides NF-κB, RAGE induces the activation of multiple intracellular pathways including mitogen-activated protein kinases (MAPKs) (Xu et al., 2010; Kim et al., 2011), nuclear factor of activated T-cells (NF-AT) (Cho et al., 2009), and the cAMP response element-binding factor (CREB) (Huttunen et al., 2002), though activation of NF-κB seems to be the main pathway in neurodegenerative conditions. This diversity suggests that there are multiple modes of RAGE activation by different ligands. Indeed, in the brain pathogenic mechanism have been attributed to different RAGE ligands, besides AGEs, including S100 proteins, amyloid peptide, lipopolysaccharide (LPS) and high mobility group box 1 (HMGB1) (Gasparotto et al., 2017).

Receptor for advanced glycation end product has indeed affinity for a large number of proteins, and new putative RAGE ligands are reported continuously. Among those, S100/calgranulin family of proinflammatory cytokine like mediators is a major inducer of RAGE activation (Hofmann et al., 1999; Donato, 2001), already reported in neurodegenerative diseases. Interestingly, S100B protein levels are increased in SNpc and CSF from PD patients (Sathe et al., 2012), and in striata from mice submitted to MPTP treatment (Viana et al., 2016). Accordingly, lack of S100B resulted in decreased expression of both RAGE and TNF-α with consequent reduced microglia activation and neuroprotection (Sathe et al., 2012), further implicating S100B/RAGE axis in the neurodegenerative process in PD. Nonetheless, S100B protein levels were unaltered in PD patients’ serum (Schaf et al., 2005), indicating that this may not be useful as a biomarker for the disease.

Another important ligand for RAGE is HMGB1, a protein with different roles during neural development and neurodegeneration. While during early brain development, HMGB1 is involved in neurite outgrowth and cell migration, during adulthood HMGB1 mediates neuroinflammation after injury (Fang et al., 2012). In PD, and other neurodegenerative diseases, HMGB1 expression is considered as an important risk factor for chronic neurodegeneration, due to its role as a pro-inflammatory mediator involved in the progression of neuroinflammation in the brain (Kim et al., 2011; Takata et al., 2012; Santoro et al., 2016). Accordingly, HMGB1 was found to be up-regulated in the cytosol of dopaminergic neurons from PD patients tissue, but not in healthy age-matched individuals (Santoro et al., 2016). This is reinforced by the detection of HMGB1 in the neuronal cytoplasm from MPTP-treated mice, together with observations that the deleterious effects induced by MPTP were partially prevented by blocking HMGB1 (Santoro et al., 2016; Sasaki et al., 2016). Due to its ability to activate RAGE, once again neutralization of HMGB1 protected dopaminergic neurons from MPTP-induced cell death by decreasing the levels of self-induced RAGE and TNF-α release (Sasaki et al., 2016). Together, these observations indicate that the downstream signaling cascades of RAGE, namely the activation of NF-κB, and the self-perpetuating expression of RAGE, contribute to the damaging effect of HMGB1 in PD.

### Glycation of α-Synuclein

As already mentioned, the major hallmark of PD is the development of Lewy bodies composed of aggregated α-synuclein (Lee and Trojanowski, 2006). Although the mechanisms underlying α-synuclein aggregation and toxicity are not fully elucidated, it is clear that its aggregation is linked with the pathogenesis of PD (Conway et al., 2000; Outeiro et al., 2009). Interestingly, α-synuclein may undergo numerous post-translational modifications, including glycation and glycosylation among others, and interacts with several endogenous and exogenous macromolecules, proteins, metals, hormones, neurotransmitters and drugs that can all interfere with its ability to form aggregates. Indeed, α-synuclein is one of the most predominantly glycated proteins in the context of PD (Vicente Miranda et al., 2017). The glycation of α-synuclein, in one of its 15 lysine residues, influences the initial formation of aggregates and induces its oligomerization, by stabilizing the formed oligomers (Padmaraju et al., 2011). As expected, AGEs were found to co-localize with α-synuclein and to accelerate its aggregation by inducing complex protein cross-links (Padmaraju et al., 2011; Guerrero et al., 2013). Oligomerization of α-synuclein is an important pathological modification, since oligomeric species are now considered to be highly toxic (Guerrero et al., 2013).

Additionally, monomeric species of glycated α-synuclein can directly interact with DNA increasing genome damage, and oligomeric and monomeric species of the glycated α-synuclein further promote the formation of ROS (Padmaraju et al., 2011; Guerrero et al., 2013). Moreover, such α-synuclein oligomers can directly affect membrane permeability by forming pores, with consequent loss of cell homeostasis and neuronal cell dysfunction (Guerrero et al., 2013). Importantly, oligomers composed of glycated α-synuclein due to protein cross-links can be resistant to degradation by the proteasome system or by other quality control systems, and thus accumulate and cause neuronal cell death. Finally, the deleterious effects of glycated α-synuclein may also arise from its ability to trigger neuroinflammation, not only by efficiently activating microglia, but also by interacting with RAGE and activating the downstream NF-κB transcription factor (Salahuddin et al., 2014). Thus, increased cell surface expression of RAGE receptors in PD allows the binding of more glycated α-synuclein, fueling a feedback loop, which sustains inflammation, α-synuclein accumulation and neuronal cell death.

### MGO in Parkinson’s Disease

The discovery that MGO can react with dopamine to forming 1-acetyl-6,7-dihydroxy-1,2,3,4-tetrahydroisoquinoline (ADTIQ), allows the establishment of another relationship between glycation and PD. This metabolite, which structure resembles that of MPTP, is found in human brain, including the SNpc, and its levels are increased in PD (Deng et al., 2012; Song et al., 2014). Therefore, up-regulated glycation in SNpc increases the probability of ADTIQ formation due to reaction with dopamine and can promote specific dopaminergic degeneration. Consistently, ADTIQ levels were increased in mouse models of familial PD, and this molecule also acted as an endogenous neurotoxin in SH-SY5Y neuroblastoma cells (Xie et al., 2015). Interestingly, the levels of ADTIQ and MGO were increased in the striatum of diabetic brains and in rat models of diabetes (Song et al., 2014; Xie et al., 2015), linking together the onset of these two pathologies.

Additionally, MGO may also interfere with the UPS, which in the context of PD, plays a major role in the clearance of misfolded α-synuclein (Ciechanover and Kwon, 2017). The failure of the UPS may arise due to the fact that ubiquitin is a target for MGO, which impairs its ability to conjugate with its target proteins (Uchiki et al., 2012). Again, this promotes a self-perpetuating reaction further contributing to α-synuclein aggregation and impairment of proteasome function, probably contributing to the deleterious effects of glycation in PD.

MGO can also directly react with mitochondria inducing the dysfunction of these organelles with concomitant increased ROS production and oxidative stress (Rosca et al., 2005; Rabbani and Thornalley, 2008; Wang et al., 2009). Interestingly, under these stress conditions cells tend to increase the rate of glycolysis in order to maintain a sufficient ATP synthesis, but as already mentioned this metabolic shift exacerbates MGO production. This mechanism may also be involved in the deleterious effects of mutated or dysfunctional PD-linked proteins like parkin and PINK1, which strongly affect mitochondrial function, ATP production and consequently energy metabolism. In fact, it has been described that loss of function mutations in parkin and PINK1 stimulate glycolysis (Vincent et al., 2012; Requejo-Aguilar et al., 2014), and therefore the propensity for an increased formation of MGO.

### Glyoxalase System

Although the formation of AGE occurs spontaneously, endogenous active mechanisms exist in cells to revert AGEing of proteins and to overcome glycation toxicity. For example, MGO is detoxified by the glyoxalase system (glyoxalase-I and -II) and by aldose reductases (Thornalley, 1993; Rabbani and Thornalley, 2012) (**Figure 2**). Glyoxalase-I is an important endogenous anti-glycation agent, that has been found to decrease with age in the human brain (Kuhla et al., 2006). Notably, another gene linked to familial PD, *PARK7*, that codes for DJ-1, has been associated with the metabolism of AGEs, through several anti-glycation activities. Although the function of DJ-1 is still not fully understood, amongst its multiple functions it was shown to modulate the activity of nuclear factor erythroid 2-related factor 2 (Nrf2), a master regulator of the cellular anti-oxidant response (Clements et al., 2006; Xue et al., 2012). Importantly, Nrf2 is an upstream key regulator of glyoxalase-I expression (Clements et al., 2006; Lee et al., 2012; Xue et al., 2012). Additionally, DJ-1 was also recently considered as a protein deglycase that restores proteins that were MGO-glycated (Richarme et al., 2015). However, controversy still exists regarding this putative direct effect of DJ-1 on MGO (Pfaff et al., 2017), instead this effect, independent of Nrf2, may be related to the ability of DJ-1 to repress glycolysis (Requejo-Aguilar et al., 2015). Additionally, Hsp-31 a member of the DJ-1 superfamily is also described as having deglycase activity (Mihoub et al., 2015). Nonetheless, loss of function mutations of DJ-1, or stress-induced DJ-1 dysfunction appear to be associated with mitochondrial impairment and increased levels of MGO, although the exact mechanism still needs to be fully understood.

In summary, increased expression of RAGE as well as RAGE activation were proposed to be involved in dopaminergic degeneration in human PD and experimental models of the disease. Moreover, several studies using experimental models of PD suggest that RAGE blocking should be beneficial in human PD. Thus, research should be conducted in order to understand the balance between the expression of sRAGE and mRAGE, in healthy individuals and PD patients, and the way the putative shifts in the expression levels of both will affect RAGE signaling. Interestingly, a decreased plasma level of sRAGE has been reported in patients with Alzheimer’s disease (AD) (Emanuele et al., 2005; Xu et al., 2017), but to our knowledge this information is lacking in PD. Nonetheless, glycation and the underlying mechanisms of RAGE activation seem to represent important therapeutic targets in PD.

## Glycosylation in Parkinson’s Disease

### Structural Diversity of Glycans

All cells and a significant number of macromolecules in our body display covalently attached sugars, named “glycans.” These glycans present a great structural variability, according to the involved building blocks (the monosaccharides) and the bond-type. In addition, alterations of the physiological condition of the cells and differentiation stage can easily alter their structures (Lowe and Marth, 2003; Haltiwanger and Lowe, 2004). The major classes are typically defined according to the nature and linkage to the conjugate, i.e., a protein or lipid (glycoprotein or glycolipid).

**FIGURE 3:**
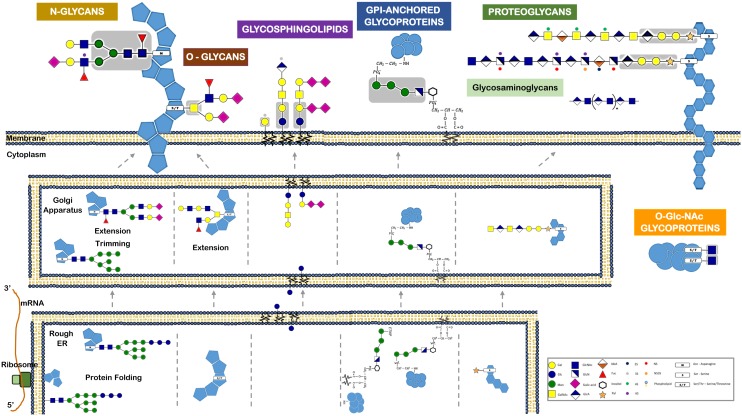
Different classes of glycans modify proteins and lipids and have distinct biosynthetic pathways. This figure refers the major glycan classes expressed by human cells and that are described in the text. It also depicts their different biosynthetic steps and their localization for initiation, trimming, and elongation within the cells. Glycans can be attached to proteins via *N*-linkage to Asparagine (N). The *N*-glycans share a common pentasaccharide core region (highlighted in the figure in a gray box) that can be further diversified. *N*-glycans are initiated by the en-bloc transfer of a large preformed precursor glycan to a newly synthesized glycoprotein. Glycans can be attached to proteins via *O*-glycosylation. Common *O*-glycans are initiated by GalNAc *O*-linked to S/T in a polypeptide chain. *O*-glycans are further extended with the sequential addition of other monosaccharides. Glycans linked to a lipid moiety ceramide are named glycolipids or glycosphingolipids. These are initiated by the addition of glucose to ceramide on the outer face of the ER-Golgi compartments, and the glycan is then flipped into the lumen to be extended. The glycosylphosphatidylinositol (GPI)-anchored proteins are glycoproteins linked to a phosphatidylinositol. Like *N*-glycans, GPI-anchored proteins are initiated by the en-bloc transfer of a large preformed precursor glycan to a newly synthesized glycoprotein. Proteoglycans are glycoproteins where glycosaminoglycans are attached to the proteins. Glycosaminoglycans are linear polymers containing disaccharide unit repeats that can be attached to proteins or exist in free form. Intracellular proteins can also be modified with *O*-GlcNAc. *O*-GlcNAcylation is the covalent link of a GlcNAc to a serine or threonine (S/T). It is distinct from all other common forms of protein glycosylation, since it occurs exclusively within the nucleus and cytoplasm and it is not further elongated or modified.

Among the various post-translational modifications that a protein may suffer, glycosylation is the most common and complex type. It is predicted that about 50–60% of human proteins, including almost all cell surface and all secreted proteins, are glycosylated (Apweiler et al., 1999; Hagglund et al., 2004; Kameyama et al., 2006). A glycoprotein usual has multiple oligosaccharide attachment sites, and each glycosylation site in turn may be altered with a variety of oligosaccharide chains (**Figure 3**).

Typically, the two major types of protein glycosylation are categorized as either *N*-glycosylation or *O*-glycosylation (**Figure 1**). *N*-glycosylation is by far the most common (Spiro, 2002; Stanley et al., 2009), involving an *N*-glycosidic bound that links the nitrogen of an asparagine residue (Asn) amide group to GlcNAc of a glycan (Nalivaeva and Turner, 2001). This Asn must belong to the consensus amino acid sequence Asn-X-Thr (X is any amino acid, excluding proline). *O*-glycosylation in humans often occurs via an N-acetylgalactosamine (GalNAc) attached to the hydroxyl group of serine or threonine residues (Spiro, 2002). The *N*-and *O*-glycosylated proteins are synthesized essentially in the ER and Golgi through sequential reactions involving several enzymes, namely sugar nucleotide synthases, transporters, glycosyltransferases, glycosidases, and other sugar-modifying enzymes. Conversely to what happens in *N*-glycosylation, *O*-glycosylation usually starts in the lumen of the ER and the assembly step is not proceeded by a remodeling step (**Figure 3**).

A distinct *O*-glycosylation is the *O*-linked N-acetylglucosamine (GlcNAc), occurring independently of the ER-Golgi pathway, and consisting in the single addition of a monosaccharide GlcNAc to nucleus and cytoplasm proteins (**Figure 3**).

Included within glycoproteins are also the proteoglycans, which are proteins that contain, through a serine residue, one or more glycosaminoglycans (GAG) chains attached. Another important glycoconjugate is the glycophosphatidylinositol (GPI) anchor, which is actually a glycan link between phosphatidylinositol and a phosphoethanolamine that is bound to the carboxy terminal of a protein. Usually this arrangement is the only anchor that attaches these type of proteins to the lipid bilayer membrane (**Figure 3**).

A glycolipid, usually designated by glycosphingolipid, is a glycan linked through glucose or galactose to the terminal primary hydroxyl group of the lipid moiety ceramide. As these ceramides consist of a long chain base (sphingosine) and a fatty acid, the glycolipids are also named glycosphingolipids (GSLs). A ganglioside, in turn, consists of an anionic glycolipid that contains one or several residues of the monosaccharide sialic acid.

### Implications of Glycosylation in Parkinson’s Disease

Glycosylation influences not only the native conformation of the protein, but also specific properties such as solubility, antigenicity and half-life, and its subcellular location. It has also a role in cell to cell communication, intracellular molecular signaling pathways, and in particular the modulation of the immune response.

Taking into account the importance of proper glycosylation in the overall functioning of proteins, it is conceivable that aberrant glycosylation may contribute to the appearance of several human pathologies. Indeed, shifts in the human serum glycoform profile in health and disease have the potential to be used as biomarkers for cancer, such as hepatocellular (Nishimura, 2011; Kamiyama et al., 2013), pancreatic (Nouso et al., 2013), renal (Hatakeyama et al., 2014), and prostate cancers (Ishibashi et al., 2014), and for other diseases, including neurodegenerative diseases (Gizaw et al., 2016). In the scope of neurodegenerative diseases, it has been recently described that Huntington’s disease transgenic mice show significantly different major glycoforms and expression levels of total glycans (Gizaw et al., 2015). Moreover, major *N*- and GSL-glycans from human AD brain, serum and CSF samples were characterized, and were shown to be different from healthy controls (Gizaw et al., 2016). Moreover, protein glycosylation was shown to be altered in human Creutzfeldt–Jakob neurodegenerative disease (Silveyra et al., 2006; Xiao et al., 2013). In the context of PD, these approaches have recently begun to emerge and are still scarce. However, the results are promising regarding the use of serum or CSF glycome profiling as a biomarker of the disease (Russell et al., 2017), as an indicator of disease stages or for defining therapeutic treatments.

Glycosylation is a major regulator of cell-to-cell and cell-environment interactions, and since the immune response relies upon countless of these contacts, it is not surprising that glycans play a major role in the immune communication and that alterations affect the patient immune status. Surprisingly, this is yet a relatively understudied and unraveled subject of glycoimmunology. Cell-surface glycans arrangements are recognized by lectins that are carbohydrate-binding receptors (Ohtsubo and Marth, 2006). These molecular recognitions between lectins and their ligands mediates complex cell-to-cell interactions, including between cells from the immune systems (van Kooyk and Rabinovich, 2008).

**FIGURE 4:**
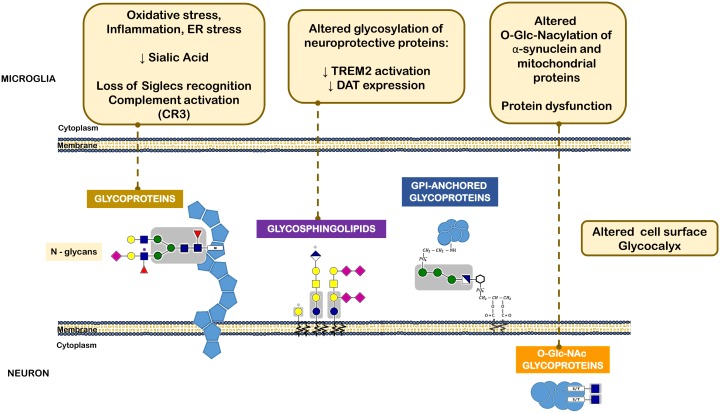
Different classes of glycans implicated in the pathogenesis of Parkinson’s disease. The oxidative stress, inflammatory milieu and ER stress reduce sialic acid content within neuronal *N*-glycans. This leads to loss of microglia Siglec receptors recognition and also complement activation. Altered glycolipids content and glycosylation of neuroprotective proteins, such as TREM2 and DAT can lead to decreased activation or expression of such proteins. *O*-GlcNAcylation of α-synuclein and other mitochondrial proteins was also observed in PD that leads to protein dysfunction.

As mentioned above, in the CNS, microglia are the immune cells that in the presence of a stimulus may manifest either pro-inflammatory or anti-inflammatory phenotypes. Microglia activation is highly regulated by carbohydrate-binding receptors such as sialic-acid-binding immunoglobulin superfamily (Siglec) and their carbohydrate ligands (Linnartz et al., 2012). Most Siglecs contain an immunoreceptor tyrosine-based inhibition motif and its signaling leads to the termination of activation signals. Thus, pro-inflammatory immune responses such as those triggered by Toll-like receptors can be turned down in microglia by inhibitory Siglec signaling (Linnartz-Gerlach et al., 2014). The terminal monosaccharides on the cell surface and extracellular proteins are frequently sialic acids. These represent the first interaction site in leukocyte communication and determine the overall immune response (Crespo et al., 2013). Sialic acid is a ligand for a number of inhibitory receptors such as Siglecs, helps masking ligands expressed by host cells from pathogen recognition and avoids autoimmune responses by inhibiting complement deposition. Consequently, alterations in sialic acid expression or in glycans decorated by sialic acids play an important role in the development of immune responses (Crespo et al., 2013). Some defects in glycosylation and, in particular, sialic acid shortage seem to exacerbate immune cell activation (Videira et al., 2008; Cabral et al., 2013; Silva et al., 2016). Pathological conditions such as oxidative stress can lead to shortage of cell surface sialic acid, probably by acidosis. In this scenario, the interactions with protective Siglecs are loss and complement components bind to cell surface and induce a complement-mediated proinflammatory cascade (**Figure 4**). Interestingly, animal models of sialic acid containing GSL (ganglioside) deficiency display some PD-like symptoms that are alleviated by administration of L-DOPA or cell-permeable ganglioside mimetics, and gangliosides may be reduced in PD patients (Wu et al., 2011). This is in agreement with a series of reports that ganglioside GM1 can alleviate symptoms in models of PD and inhibit aggregation of α-synuclein (Schneider et al., 1995; Martinez et al., 2007).

As another example, a recent report showed that altered glycosylation is also evident in peripheral immunoglobulin (IgG) glycome, with patients carrying a more pro-inflammatory fraction of IgG (Russell et al., 2017). These pro-inflammatory modifications in the peripheral IgG glycome may sustain a low-grade inflammation, triggering a positive feed-back loop that may maintain and spread the inflammation. In the context of a progressive degenerative disease associated with neuroinflammation like PD, this loop may lead to BBB disruption (Kortekaas et al., 2005; Mosley et al., 2012; Sweeney et al., 2018), enabling molecular transfer from peripheral blood. This could further explain the relationship between the peripheral alterations in the glycome and exacerbation and spreading of CNS pathogenic mechanisms.

Additionally, changes in glycosylation forms and regulation of the amounts of glycans expressed in cells, either in CNS or periphery, can be influenced by the accumulation α-synuclein. Interestingly, α-synuclein has been found in body fluids, including blood and CSF, and is likely produced by both peripheral tissues and the CNS (Sui et al., 2014). Exchange of α-synuclein between the brain and peripheral tissues is mainly due to increased BBB permeability and can have important pathophysiologic implications, such as modulation of glycans assembly. Additionally, in the course of PD progression RAGE levels at the BBB are increased, as well as AGE and RAGE levels in CNS cells (Dalfó et al., 2005; Ding and Keller, 2005; Kurz et al., 2011; Sathe et al., 2012), with subsequent increased neuronal death and abnormal sugar metabolism. This can decrease the concentration of brain type specific *N*-glycans and gangliosides, linking the deleterious effects of glycation and aberrant glycosylation.

We still know very little about the role of glycosylation of specific proteins in PD, although it appears that the glycome may be different between patients and age-matched healthy individuals (Russell et al., 2017). Interestingly, two putative *N*-glycosylation sites were found in the V-type ligand domain of mature RAGE protein (Neeper et al., 1992; Kislinger et al., 1999). While the structural/functional basis underlying the ability of RAGE to bind a multitude of ligands was not revealed yet, one possibility is that these *N*-glycosylation sites may influence RAGE signaling, by modulating its binding to different molecules, and thus modifying the intracellular pathways activated by this receptor in different cellular contexts.

Another example of a PD-related protein that may be subjected to impaired glycosylation is the surface receptor triggering receptor expressed on myeloid cells 2 (TREM2), a type I membrane protein expressed on myeloid cells including microglia. TREM2 has multiple ligands, including Apolipoprotein E (APOE), lipids, and seems to transduce its signal through interaction with DNAX-activating protein. Activation of TREM2 was suggested to have an anti-inflammatory function after ligand binding in various diseases, including PD (Rayaprolu et al., 2013). Interestingly, *N*-glycans including sialylated and/or fucosylated complex-type glycans can decorate TREM2, and alterations in a portion of these *N*-glycans can dictate the conformation or trafficking of TREM2, leading to altered protein stability and impaired antioxidant potential (Park et al., 2015) (**Figure 4**).

Shimura et al. (2001) found post translationally modified form of human α-synuclein containing *O*-linked sugars. Interestingly, whereas normal parkin recognizes and binds to glycosylated α-synuclein, they showed that in PD mutant parkin was not able to bind the *O*-glycosylated α-synuclein. Therefore, this suggests that this mechanism is involved in the accumulation of ubiquitinated α-synuclein in Lewy bodies in familiar but also in idiopathic PD, where loss of parkin function was already described (Shimura et al., 2001).

It has been then shown that α-synuclein, similarly to several aggregation-prone proteins that directly contribute to neurodegeneration are modified by a specific *O*-glycan, the *O*-GlcNAcylation (Alfaro et al., 2012). *O*-GlcNAcylation has been shown to affect the phosphorylation of α-synuclein and block the toxicity of α-synuclein, suggesting that increasing *O*-GlcNAcylation may prevent protein aggregation (Marotta et al., 2015) (**Figure 4**).

As discussed above, mitochondria dysfunction plays a central role in the neurodegenerative process in PD. However, although the glycosylation process involved in secreted, membrane and nucleocytosolic proteins is substantially well-understood, this is not yet the case for mitochondrial proteins. So far there are only a scarce studies on glycosylated mitochondrial proteins (Levrat et al., 1989; Chandra et al., 1998; Hu et al., 2009; Kung et al., 2009), but together suggest that mitochondrial glycome may dictate mitochondrial function and cell fate. A number of mitochondrial proteins are predicted as a target of glycosylation, and these glycosylated isoforms, could be a still unexplored mechanism for regulating mitochondrial metabolic functions even if they represent just a small fraction of the total expressed proteins in these organelles (Burnham-Marusich and Berninsone, 2012). Accordingly, Zhao et al. (2014) showed that enhanced *O*-GlcNAcylation of mitochondrial proteins might protect from oxidative stress and increase mitochondrial respiration rate in aged retina. Whereas others indicate that *O*-GlcNAcylation, might contribute to impaired mitochondrial function. Although the relationship between *O*-GlcNAcylation and mitochondria is not yet completely characterized, it is suggested that *O*-GlcNAcylation could mediate the link between mitochondrial motility and availability of mitochondrial substrates in neurons (Pekkurnaz et al., 2014). Importantly, the master regulator of mitochondrial biogenesis PGC1-α (Wang X. et al., 2016) and ATP synthase α-subunit (Kung et al., 2009) were shown to be *O*-GlcNAcylated, however, the physiological role is not very clear yet. Therefore, one may extrapolate that these mechanisms could also be recapitulated in PD or other neurodegenerative diseases, and that abnormal mitochondrial glycome could trigger the acceleration of mitochondrial dysfunction and neuronal death. In fact, *O*-GlcNAcylation in lysates from the *post-mortem* temporal cortex of PD patients was shown to be detrimental to neurons by inhibition of autophagy and with consequent α-synuclein accumulation (Wani et al., 2017).

Although the causes of idiopathic PD are still unknown, dopamine metabolism may be an important source of ROS in dopaminergic cells. The cytosolic levels of dopamine depend on its biosynthesis, and on two transporters, namely the cell membrane dopamine transporter (DAT) and vesicular monoamine transporter (VMAT). Dopamine uptake from the synaptic cleft is undertaken by DAT, a glycoprotein with 12-transmembrane domains, which interestingly is also required for the uptake of neurotoxins like MPTP that are similar to dopamine (Storch et al., 2004). The human DAT is a heavily glycosylated protein containing three putative *N*-linked glycosylation sites in the second extracellular (Li et al., 2004). Notably, the *N*-linked glycosylation status of DAT influences its cell surface expression and transporter activity (Hastrup et al., 2001; Torres et al., 2003). In fact, the expression of DAT on the membranes can be efficiently inhibited by tunicamycin or mutations of its *N*-linked glycosylation sites (Hastrup et al., 2001). Additionally, glycosylation is also crucial for DAT activity, since dopamine is more efficiently transported by glycosylated DAT than by the non-glycosylated form of the transporter (Torres et al., 2003; Li et al., 2004).

Thus, an aberrant glycosylation may contribute to a decrease in DAT membrane expression, as well as an imbalance between the functional vs. dysfunctional (or less efficient) receptor populations. Both processes result in an accumulation of dopamine in the extramembrane space, thus contributing to an increase in ROS formation in the vicinity of dopaminergic neurons. Interestingly, parkin can significantly enhance the ubiquitination of misfolded DAT that results from by *N*-glycosylation inhibition by tunicamycin, consequently attenuating the damaging effects of aberrantly glycosylated DAT on dopamine uptake (Jiang et al., 2004). Accordingly to what was found for α-synuclein, normal parkin, but not its PD-linked T240R mutant form, can recover DAT membrane expression and transporter activity even in the presence of an abnormal glycosylated form of the protein (Jiang et al., 2004). These results highlight the neuroprotective role of native parkin, through a less well-known mechanism that involves its ability to maintain proper dopamine uptake. Parkin, in fact, ubiquitinates and targets for degradation misfolded abnormally glycosylated DAT, and favors the proper membrane localization of this transporter, contributing to an efficient dopamine recycling and reduction of dopamine-associated toxicity toward neighboring cells.

In summary, about one-third of the cellular proteins are synthesized in the ER where they gain proper folding and undergo posttranslational modifications to become mature functional peptides, and then move on to their target organelle or are secreted. Amongst all the proteins that are synthesized most of them are glycosylated at the ER, in a cascade of reactions that involve an orchestrated activation of several different enzymes. Since ER stress is one of the identified pathological mechanisms in PD (Mercado et al., 2013; Tsujii et al., 2015), aberrant glycosylation might indeed be due to an overload of the ER with underglycosylated proteins. Additionally, oxidative stress and inflammation may also trigger abnormal glycosylation in PD. The above mentioned mechanisms are depicted in **Figure 4**.

## Conclusion

Here, we focused on the established concepts of neuroinflammation and abnormal glucose metabolism in PD, linking them to mitochondrial dysfunction and emerging fields of research that take into account current discoveries suggesting that glycation and abnormal glycosylation may underlie the development and/or progression of this disease.

**FIGURE 5:**
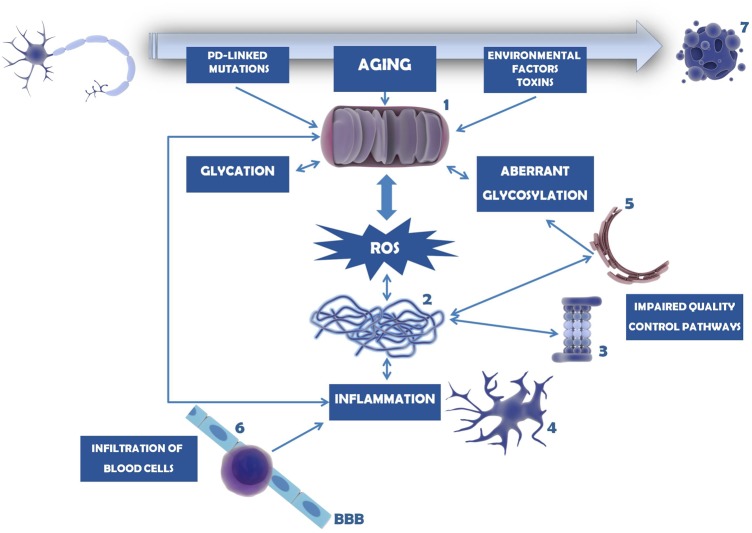
Mechanisms involved in dopaminergic neuron cell death in PD. Mitochondria dysfunction in PD can be caused by mutations in PD-linked proteins (e.g., parkin, PINK1, DJ-1), environmental factors and toxins (e.g., MPTP, 6-OHDA, pesticides, herbicides) or age-induced depletion of endogenous antioxidant defense mechanisms (1). As a consequence, ROS levels and oxidative stress increase and accumulate, overcoming the endogenous antioxidant capacity. Mitochondria dysfunction and oxidative stress may trigger glycation that in turn will affect several proteins, including mitochondrial proteins. Under these circumstances, proteins, in particular α-synuclein, tend to be misfolded and to form insoluble aggregates (2). This is further potentiated by impairment of several quality control pathways in PD, namely proteasome-dependent protein degradation (3), UPR, UPS, and autophagy. Protein aggregates and oxidative stress activate microglia and trigger the neuroinflammatory response within the brain (4). Inflammation, malfunction of ER resident enzymes responsible for glycosylation of target proteins (5), among other causes, may induce aberrant protein glycosylation. Impairment of this mechanism may underlie improper protein function as well as abnormal cell–cell contacts. With PD progression BBB permeability increases and peripheral T cells invade the CNS (6), contributing to the accumulation of pro-inflammatory mediators and further activating microglia. In turn, (neuro)inflammation may damage mitochondria, entering a vicious cycle of deleterious effects that ultimately lead to dopaminergic cell death (7).

Mitochondria are key organelles in the development and progression of PD. Not only they are the major source or ROS within the cells, as they are also very susceptible to ROS-induced effects. Several different causes and pathways may affect mitochondria integrity and function, with severe consequences in the assembly of proteins, and induction of (neuro)inflammation. These mechanisms seem to be linked to each other in a circular way, since more oxidative stress generates more inflammation and *vice versa*, as briefly schematized in **Figure 5**.

Understanding the glycosylation pattern of glycoproteins which are affected by either genetic or environmental cellular stressors in PD, can be a promising approach for the discovery of novel biomarkers to assist an easy prognosis. However, only a few glyco-PD studies have been performed, and our knowledge of glycan functions in the context of PD is still limited. The characterization of the general *N*-glycome as well as specific glycosylated membrane proteins in peripheral blood cells from PD patients and healthy controls will reveal the potential contribution of aberrant glycosylation in the cellular dysfunctions leading to neurodegeneration in PD. Certainly, the large-scale serum glycomics of a variety of stages of PD patients could accelerate the discovery of novel class of biomarkers and molecular targets toward the development of the diagnostic and therapeutic agents for this disease.

## Author Contributions

PV and MC-C wrote the manuscript, and approved the submitted version.

## Conflict of Interest Statement

The authors declare that the research was conducted in the absence of any commercial or financial relationships that could be construed as a potential conflict of interest.

## Abbreviations

AD: Alzheimer’s disease
ADTIQ: 1-acetyl-6,7-dihydroxy-1,2,3,4-tetrahydroisoquinoline
APC: antigen presenting cell
ATF6: activating transcription factor-6
AGE: advanced glycation end-product
BBB: blood–brain barrier
CHOP: C/EBP-homologous protein
CNS: central nervous system
COX-2: cyclooxygenase-2
CREB: cAMP response element-binding factor
CSF: cerebrospinal fluid
DAT: dopamine transporter
ER: endoplasmic reticulum
ERAD: ER-associated degradation
GAG: glycosaminoglycan
GalNAc: *N*-acetylgalactosamine
GlcNAc: *N*-acetylglucosamine
GPI: glycophosphatidylinositol
GRP78: glucose-regulated protein-78
GSLs: glycosphingolipids
HMGB1: high mobility group box 1
IL-1β: interleukin-1 beta
IFN-γ: interferon-gamma
IgG: immunoglobulin G
iNOS: inducible nitric oxide synthase
IRE1: inositol requiring enzyme-1
LPS: lipopolysaccharide
MAPK: mitogen-activated protein kinase
MGO: methylglyoxal
MHC: major histocompatibility complex
MPP^+^: 1-methyl-4-phenylpyridinium
MPTP: 1-methyl-4-phenyl-1,2,3,6-tetrahydropyridine
mtDNA: mitochondrial DNA
NKT: natural killer T
NF-AT: nuclear factor of activated T cells
NF-κB: nuclear factor-kappa B
NO: nitric oxide
Nrf2: nuclear factor erythroid 2-related factor 2
6-OHDA: 6-hydroxydopamine
PARIS: parkin interacting substrate
PERK: protein kinase RNA-like ER kinase
PD: Parkinson’s disease
PGC-1α: peroxisome proliferator-activated receptor gamma coactivator-1α
PINK 1: protein phosphatase and tensin homolog (PTEN)-induced putative kinase 1
RAGEs: receptor for advanced glycation end products
ROS: reactive oxygen species
Siglec: sialic-acid-binding immunoglobulin superfamily
SNpc: *Substantia nigra pars compacta*
TNF-α: tumor necrosis factor alpha
TREM2: triggering receptor expressed on myeloid cells 2
UPR: unfolded protein response
UPS: ubiquitin–proteasome system
VMAT: vesicular monoamine transporter
